# Circulating Memory T Follicular Helper Cells in Patients with Neuromyelitis Optica/Neuromyelitis Optica Spectrum Disorders

**DOI:** 10.1155/2016/3678152

**Published:** 2016-03-09

**Authors:** Xueli Fan, Yanfang Jiang, Jinming Han, Jingyao Liu, Yafen Wei, Xinmei Jiang, Tao Jin

**Affiliations:** ^1^Department of Neurology and Neuroscience Center, The First Hospital of Jilin University, Jilin University, Changchun 130000, China; ^2^Genetic Diagnosis Center, The First Hospital of Jilin University, Jilin University, Changchun 130000, China; ^3^Key Laboratory for Zoonosis Research, Ministry of Education, The First Hospital of Jilin University, Jilin University, Changchun 130000, China; ^4^Jiangsu Co-Innovation Center for Prevention and Control of Important Animal Infectious Diseases and Zoonosis, Yangzhou 225000, China; ^5^Department of Neurology, The Hospital of Heilongjiang Province, Harbin 150000, China

## Abstract

*Objective*. This study aimed to examine the potential role of memory T follicular helper (Tfh) cells in patients with neuromyelitis optica/neuromyelitis optica spectrum disorders (NMO/NMOSD).* Methods*. The percentages of different subsets of circulating memory Tfh cells in 25 NMO/NMOSD patients before and after treatment as well as in 17 healthy controls were examined by flow cytometry. The levels of IL-21 and AQP4 Ab in plasma and CSF were measured by ELISA.* Results*. The percentages and numbers of circulating memory Tfh cells, ICOS^+^, CCR7^−^, CCR7^−^ICOS^+^, CCR7^+^, CCR7^+^ICOS^+^ memory Tfh cells, and the levels of IL-21 in plasma and CSF were significantly increased in NMO/NMOSD patients. The percentages of CCR7^−^ and CCR7^−^ICOS^+^ memory Tfh cells were positively correlated with ARR, plasma IL-21, and AQP4 Ab levels. The percentages of CCR7^+^ and CCR7^+^ICOS^+^ memory Tfh cells were positively correlated with CSF white blood cell counts, proteins, and IL-21 levels. Treatment with corticosteroids significantly reduced the numbers of CCR7^−^ICOS^+^ and CCR7^+^ICOS^+^ memory Tfh cells as well as plasma IL-21 levels in patients with partial remission.* Conclusions*. Our findings indicate that circulating memory Tfh cells may participate in the relapse and development of NMO/NMOSD and may serve as a new therapeutic target.

## 1. Introduction

After a primary response, most effector T cells undergo apoptosis, whereas a small proportion survive and become memory T cells [1]. Memory T cells induce a potent and quick secondary response upon antigen rechallenge and participate in the pathogenesis of recurrent autoimmune diseases [1, 2]. Memory T cells consist of two distinct subsets, central memory (chemokine receptor 7-positive [CCR7^+^] memory) T cells, which lack immediate effector function but migrate to secondary lymphoid tissues where they proliferate and differentiate into effector cells, and effector memory (CCR7^−^ memory) T cells, which migrate to inflamed peripheral tissues and display immediate effector function [3].

Neuromyelitis optica (NMO) is an autoimmune disease characterized by severe optic neuritis and transverse myelitis [4]. NMO spectrum disorders (NMOSD) are limited forms of NMO, including optic-spinal multiple sclerosis, relapsing isolated optic neuritis, recurrent transverse myelitis, and optic neuritis or myelitis in the context of certain autoimmune diseases [4]. The etiology and mechanisms underlying the development and relapse of NMO/NMOSD are not completely understood. It is generally accepted that NMO/NMOSD are complicated immunological disorders mainly involving humoral immunity [5, 6]. Aquaporin 4 (AQP4) antibody (Ab) plays a crucial role in the pathogenesis of NMO/NMOSD [7]. Approximately 90% of patients with NMO and more than 50% of patients with NMOSD are positive for AQP4 Ab [8]. It is proposed that AQP4 Ab binding to AQP4, which is the predominant water channel expressed in the astrocytic foot processes of the central nervous system (CNS), leads to complement activation, blood-brain barrier disruption, astrocytic membrane damage, oligodendrocyte death, myelin loss, and neuronal injury [7, 9, 10]. NMO/NMOSD are usually followed by relapse and a poor prognosis [4]. A recent study showed that memory Th17 cells are involved in the development and relapse of NMO, whereas intravenous methylprednisolone reduces memory Th17 proportions [2]. However, how memory T cell immunity regulates humoral immunity during relapse in NMO/NMOSD patients has not been clarified.

T follicular helper (Tfh) cells play a key role in humoral immunity. These cells are defined by their expression of the transcription factor B cell lymphoma-6 (Bcl-6), the cell surface markers CXC-chemokine receptor 5 (CXCR5), inducible costimulator (ICOS), programmed death-1 (PD-1), and CD40 ligand (CD40L) as well as the secretion of interleukin-21 (IL-21) [11]. Dysregulation of Tfh cell generation and function causes autoimmunity [12]. It has been demonstrated that Tfh cells may participate in pathogenetic autoantibody production in systemic lupus erythematosus [13, 14], rheumatoid arthritis [15], ankylosing spondylitis [16], and primary Sjögren's syndrome [17]. Tfh cells yield a population of cells that emigrate from the germinal center (GC) of lymphoid tissues and return to the circulation as a population of quiescent memory-type CD4^+^CXCR5^+^ T cells [18]. Most circulating Tfh cells are able to provide support for the differentiation of naive and memory B cells into Ab-producing cells through IL-21, IL-10, ICOS, and cognate interaction with B cells* in vitro* [19]. Therefore, circulating memory Tfh cells serve as potential biomarkers for monitoring dysregulated Ab responses in autoimmune diseases [19, 20]. To date, the role of memory Tfh cells in the pathogenesis of NMO/NMOSD is unclear. Little is known about how different subsets of circulating memory Tfh cells exist in NMO/NMOSD patients and whether different subsets of circulating memory Tfh cells are associated with relapse of NMO/NMOSD. High-dose intravenous methylprednisolone is the routine therapy for NMO/NMOSD during relapse [21]. It is also unclear how methylprednisolone affects different subsets of memory Tfh cells in patients with NMO/NMOSD.

Hence, in our study, we investigated the percentages and numbers of different subsets of circulating memory Tfh cells in NMO/NMOSD patients before and after treatment. The levels of IL-21 and AQP4 Ab in plasma and cerebrospinal fluid (CSF) also were examined. Moreover, we explored the potential relationships among values of these measures and clinical outcomes to clarify the potential roles of different subsets of memory Tfh cells in the relapse of NMO/NMOSD.

## 2. Methods

### 2.1. Patients and Controls

Written informed consent was obtained from all individual participants. The study was approved by the Medical Ethics Committee of the First Hospital of Jilin University, Changchun, China. Twenty-five patients with relapsed NMO/NMOSD were enrolled from the inpatient service of the Department of Neurology, the First Hospital of Jilin University (Changchun, China), from July 2014 to June 2015. These patients fulfilled either the Wingerchuk criteria 2006 for NMO [22] or the diagnostic criteria for NMOSD [4]. Among these patients, relapse was defined as a sudden appearance of new neurological symptoms and signs, or worsening of existing symptoms, lasting for at least 24 hours. No patients had received corticosteroid or immunosuppressant therapy in the 4 weeks prior to their enrollment in this study. Two patients had other autoimmune diseases. The disease severity of individual patients was assessed by the Expanded Disability Status Scale (EDSS). We also recruited 17 age- and gender-matched healthy controls (HCs) through the Physical Examination Center of the hospital. Their demographic and clinical characteristics are shown in Table 1. Among the NMO/NMOSD patients, 15 patients received a lumbar puncture. Furthermore, we also enrolled 8 age- and gender-matched patients with noninflammatory neurological diseases (NNDs) who received a lumbar puncture as controls. The demographic and clinical features of NMO/NMOSD and NND patients are shown in Table 2.

### 2.2. Treatment and Follow-Up

After enrollment in this study, all patients were treated with corticosteroids (pulse methylprednisolone 1000 mg for 5 days followed by gradual tapering). The patients visited the outpatient office 4–8 weeks after treatment for the follow-up. A total of 12 patients returned, and their clinical characteristics are shown in Table 3.

### 2.3. Blood and CSF Sampling and Analyses

We collected fasting venous blood samples from individual HCs and NMO/NMOSD patients before and 4–8 weeks after treatment. One part of each blood sample was centrifuged to prepare plasma samples. The remaining blood was used to prepare peripheral blood mononuclear cells (PBMCs) via density-gradient centrifugation using Lymphoprep (Axis-Shield PoC AS, Oslo, Norway). In addition, we collected CSF samples from 15 NMO/NMOSD patients and 8 NND patients when they underwent a lumbar puncture. CSF samples containing blood were excluded. The numbers of white blood cell (WBCs) and lymphocytes in peripheral blood, as well as CSF WBC counts, CSF protein levels, and CSF immunoglobulin G (IgG) levels, were routinely examined in the hospital.

### 2.4. Flow Cytometric Analysis (FCM)

Human PBMCs at 10^6^/tube were stained in duplicate with allophycocyanin (APC)-H7-anti-CD3, BV510-anti-CD4, fluorescein isothiocyanate (FITC)-anti-CD45RA, phycoerythrin (PE)-Cy*™*7-anti-CCR7, peridinin-chlorophyll proteins (PerCP)-Cy*™*5.5-anti-CXCR5, PE-anti-ICOS, BV421-anti-PD-1, PE-CF594-anti-CD154, or proper IgG isotype controls (Becton Dickinson, San Diego, CA, USA) at room temperature for 30 minutes. After being washed with phosphate-buffered saline (PBS), the cells were analyzed by flow cytometric analysis using a BD FACSAria*™* II (BD Biosciences, San Jose, CA, USA). The data were analyzed with FlowJo software (version 7.6.2, by Flowjo LLC, OR, USA). We analyzed at least 50,000 events per sample and calculated the numbers of different subsets of circulating memory Tfh cells in individual samples according to the counts of lymphocytes per liter of blood multiplied by the percentage of different subsets of memory Tfh cells in lymphocytes.

### 2.5. Indirect Immunofluorescence Test (IIFT)

The serostatus of AQP4 Ab in all patients was measured through IIFT systems according to the manufacturer's instructions (Euroimmun Medizinische Labordiagnostika, Lubeck, Germany).

### 2.6. Enzyme-Linked Immunosorbent Assay (ELISA)

The levels of plasma and CSF IL-21 were measured by ELISA kits according to the manufacturer's instructions (Multi Sciences Biotech Co., Hangzhou, China). The detection limit for human IL-21 was 11.99 pg/mL. The levels of plasma and CSF AQP4 Ab were measured by ELISA using a specific kit (Yuanye Bio-Technology Co., Shanghai, China) in AQP4 Ab-seropositive patients. The sensitivity of this assay was 1.0 ng/mL.

### 2.7. Statistical Analysis

Data are expressed as medians and ranges. Differences between HCs and NMO/NMOSD patients were analyzed by Mann-Whitney *U* nonparametric tests, and differences between NMO/NMOSD patients before and after treatment were analyzed by Wilcoxon tests. The relationship between variables was evaluated by the Spearman rank correlation test. Statistical analyses were performed using SPSS 19.0 software (SPSS, Inc., Chicago, IL, USA), and statistical significance was determined according to a two-sided *P* value <0.05.

## 3. Results

### 3.1. Circulating Memory Tfh Cells in NMO/NMOSD Patients and HCs

**Figure 1:**
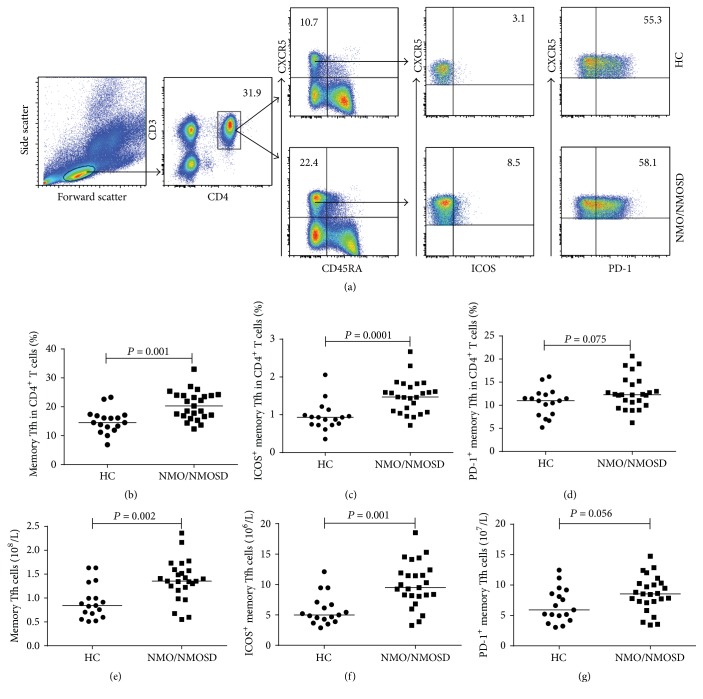
FACS analysis of circulating memory Tfh cells and ICOS^+^ memory Tfh cells in individual participants. PBMCs were isolated from individual participants and stained with different fluorescent antibodies. The cells were gated on lymphocytes, CD3^+^ and CD4^+^, and then CXCR5^+^ and CD45RA^−^ cells. The percentage of CD3^+^CD4^+^CXCR5^+^CD45RA^−^ (memory) Tfh cells was determined. Subsequently, memory Tfh cells were gated on ICOS. We analyzed the percentages of memory Tfh and ICOS^+^ memory Tfh cells in lymphocytes and calculated the numbers of each subtype of cells in total lymphocytes per liter. (a) Flow cytometric analysis. ((b)-(c)) The percentages of memory Tfh cells and ICOS^+^ memory Tfh cells in HCs and untreated NMO/NMOSD patients. ((d)-(e)) The numbers of memory Tfh cells and ICOS^+^ memory Tfh cells in HCs and untreated NMO/NMOSD patients. The horizontal lines indicate the median values for each group.

**Figure 2:**
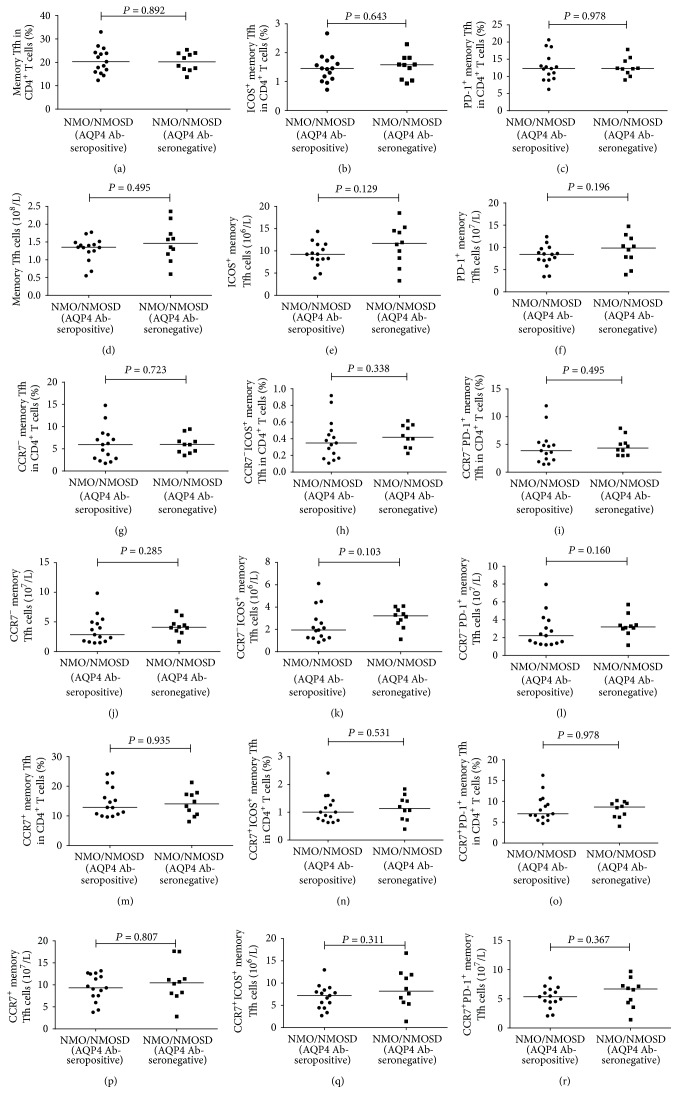
Analysis of the percentages and numbers of different subsets of memory Tfh, CCR7^−^ memory Tfh, and CCR7^+^ memory Tfh cells in AQP4 Ab-seropositive and AQP4 Ab-seronegative patients. ((a)–(c)) The percentages of memory Tfh, ICOS^+^ memory Tfh, and PD-1^+^ memory Tfh cells in AQP4 Ab-seropositive and AQP4 Ab-seronegative patients. ((d)–(f)) The numbers of memory Tfh, ICOS^+^ memory Tfh, and PD-1^+^ memory Tfh cells in AQP4 Ab-seropositive and AQP4 Ab-seronegative patients. ((g)–(i)) The percentages of CCR7^−^, CCR7^−^ICOS^+^, and CCR7^−^PD-1^+^ memory Tfh cells in AQP4 Ab-seropositive and AQP4 Ab-seronegative patients. ((j)–(l)) The numbers of CCR7^−^, CCR7^−^ICOS^+^, and CCR7^−^PD-1^+^ memory Tfh cells in AQP4 Ab-seropositive and AQP4 Ab-seronegative patients. ((m)–(o)) The percentages of CCR7^+^, CCR7^+^ICOS^+^, and CCR7^+^PD-1^+^ memory Tfh cells in AQP4 Ab-seropositive and AQP4 Ab-seronegative patients. ((p)–(r)) The numbers of CCR7^+^, CCR7^+^ICOS^+^, and CCR7^+^PD-1^+^ memory Tfh cells in AQP4 Ab-seropositive and AQP4 Ab-seronegative patients.

We measured the percentages and numbers of different subsets of circulating memory Tfh cells (CD3^+^CD4^+^CXCR5^+^CD45RA^−^ T cells) in NMO/NMOSD patients before and after treatment as well as in HCs (Figure 1(a)). Both the percentages of memory Tfh cells and ICOS^+^ memory Tfh cells among CD4^+^ T cells were significantly greater in NMO/NMOSD patients before treatment than those in the HC group (*P* = 0.001 and *P* = 0.0001, resp.; Figures 1(b) and 1(c)). In line with these results, the numbers of circulating memory Tfh cells and ICOS^+^ memory Tfh cells were significantly higher in patients than in HCs (*P* = 0.002 and *P* = 0.001, resp.; Figures 1(e) and 1(f)). In contrast, there were no significant differences in the percentages and numbers of PD-1^+^ memory Tfh cells between the patients and HCs (*P* = 0.075 and *P* = 0.056, resp.; Figures 1(d) and 1(g)). Furthermore, we measured the serostatus of AQP4 Ab in all NMO/NMOSD patients and found that 15 of 25 patients were AQP4 Ab-seropositive. We compared the percentages and numbers of different subsets of memory Tfh cells in AQP4 Ab-seronegative patients and AQP4 Ab-seropositive patients and found no difference (Figures 2(a)–2(f)).

### 3.2. Circulating CCR7^−^ Memory Tfh Cells in NMO/NMOSD Patients and HCs

**Figure 3:**
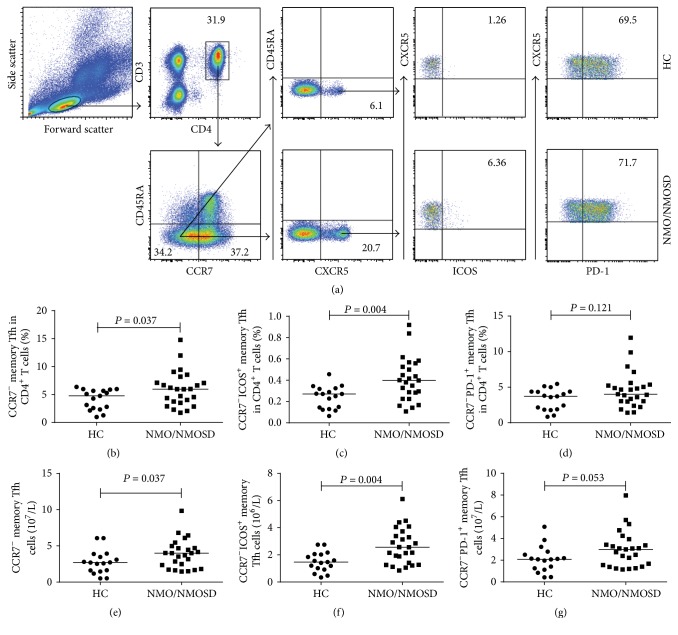
FACS analysis of circulating CCR7^−^ and CCR7^−^ICOS^+^ memory Tfh cells in individual participants. After staining with different fluorescent antibodies, the cells were gated sequentially on lymphocytes, CD3^+^ and CD4^+^, and then CD45RA^−^ and CCR7^−^ cells. After that, the cells were gated on CXCR5 and we obtained CD3^+^CD4^+^CD45RA^−^CCR7^−^CXCR5^+^ (CCR7^−^ memory Tfh) cells. Subsequently, CCR7^−^ memory Tfh cells were gated on ICOS expression, and the percentages of CCR7^−^ and CCR7^−^ICOS^+^ memory Tfh cells were analyzed by flow cytometry. The numbers of each subtype of cells were calculated. (a) Flow cytometric analysis. ((b)-(c)) The percentages of circulating CCR7^−^ and CCR7^−^ICOS^+^ memory Tfh cells in HCs and untreated NMO/NMOSD patients. ((d)-(e)) The numbers of circulating CCR7^−^ and CCR7^−^ICOS^+^ memory Tfh cells in HCs and untreated NMO/NMOSD patients. The horizontal lines indicate the median values for each group.

We then measured the percentages and numbers of different subsets of circulating CCR7^−^ memory Tfh cells (Figure 3(a)). We found that the percentages of CCR7^−^ and CCR7^−^ICOS^+^ memory Tfh cells among CD4^+^ T cells in untreated NMO/NMOSD patients were significantly higher than those in the HC group (*P* = 0.037 and *P* = 0.004, resp.; Figures 3(b) and 3(c)). The cell counts of these CCR7^−^ and CCR7^−^ICOS^+^ memory Tfh cells were also greater in patients than in HCs (*P* = 0.037 and *P* = 0.004, resp.; Figures 3(e) and 3(f)). However, there were no significant differences in the percentages and numbers of CCR7^−^PD-1^+^ memory Tfh cells between the patients and HCs (*P* = 0.121 and *P* = 0.053, resp.; Figures 3(d) and 3(g)). Furthermore, there were no differences in the percentages and numbers of different subsets of CCR7^−^ memory Tfh cells between AQP4 Ab-seronegative and AQP4 Ab-seropositive patients (Figures 2(g)–2(l)).

### 3.3. Circulating CCR7^+^ Memory Tfh Cells in NMO/NMOSD Patients and HCs

**Figure 4:**
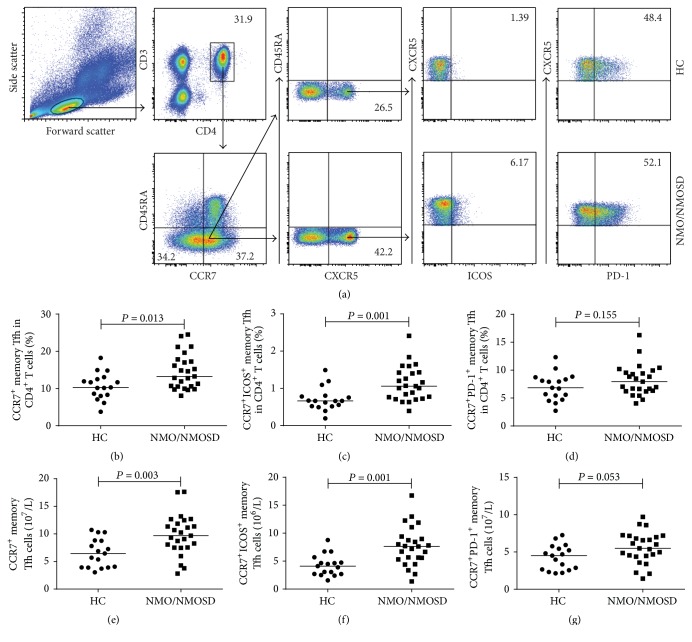
FACS analysis of circulating CCR7^+^ and CCR7^+^ICOS^+^ memory Tfh cells in individual participants. After staining with different fluorescent antibodies, the cells were gated sequentially on lymphocytes, CD3^+^ and CD4^+^, and then CD45RA^−^ and CCR7^+^ cells. After that, the cells were gated on CXCR5, and we obtained CD3^+^CD4^+^CD45RA^−^CCR7^+^CXCR5^+^ (CCR7^+^ memory Tfh) cells. Subsequently, CCR7^+^ memory Tfh cells were gated on ICOS expression, and the percentages of CCR7^+^ and CCR7^+^ICOS^+^ memory Tfh cells were analyzed by flow cytometry. The numbers of each subtype of cells were calculated. (a) Flow cytometric analysis. ((b)-(c)) The percentages of circulating CCR7^+^ and CCR7^+^ICOS^+^ memory Tfh cells in HCs and untreated NMO/NMOSD patients. ((d)-(e)) The numbers of circulating CCR7^+^ and CCR7^+^ICOS^+^ memory Tfh cells in HCs and untreated NMO/NMOSD patients. The horizontal lines indicate the median values for each group.

Next, we measured the percentages and numbers of different subsets of circulating CCR7^+^ memory Tfh cells in patients and HCs (Figure 4(a)). The percentages of CCR7^+^ and CCR7^+^ICOS^+^ memory Tfh cells among CD4^+^ T cells were significantly higher in untreated NMO/NMOSD patients than in HCs (*P* = 0.013 and *P* = 0.001, resp.; Figures 4(b) and 4(c)). Similarly, the numbers of CCR7^+^ and CCR7^+^ICOS^+^ memory Tfh cells were significantly higher in patients than in HCs (*P* = 0.003 and *P* = 0.001, resp.; Figures 4(e) and 4(f)). Nevertheless, there were no significant differences in the percentages and numbers of CCR7^+^PD-1^+^ memory Tfh cells between the NMO/NMOSD patients and HCs (*P* = 0.155 and *P* = 0.053, resp.; Figures 4(d) and 4(g)). Moreover, there were no differences in the percentages and numbers of different subsets of CCR7^+^ memory Tfh cells between AQP4 Ab-seronegative and AQP4 Ab-seropositive patients (Figures 2(m)–2(r)).

### 3.4. Levels of Plasma and CSF IL-21 in NMO/NMOSD Patients, NND Patients, and HCs

**Figure 5:**
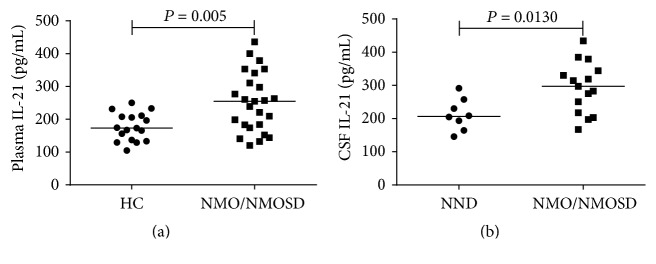
Analysis of plasma and CSF IL-21 in NMO/NMOSD patients. The levels of plasma and CSF IL-21 were measured by ELISA. (a) The levels of plasma IL-21 in NMO/NMOSD patients and HCs. (b) The levels of CSF IL-21 in NMO/NMOSD patients and NND patients. Data are expressed as mean values for individual samples. The horizontal lines represent the median values.

We measured the levels of soluble IL-21 in plasma from all participants. In addition, the levels of IL-21 in CSF samples from 15 NMO/NMOSD patients and 8 NND patients who underwent a lumbar puncture were also measured by ELISA. The results showed that levels of soluble IL-21 were higher in untreated NMO/NMOSD patients than in HCs (*P* = 0.005; Figure 5(a)). Moreover, the levels of CSF IL-21 were also greater in the NMO/NMOSD patients than in the NND patients (*P* = 0.0130; Figure 5(b)).

### 3.5. Correlations between Different Subsets of Circulating Memory Tfh Cells and Values of Clinical Measures in NMO/NMOSD Patients

**Figure 6:**
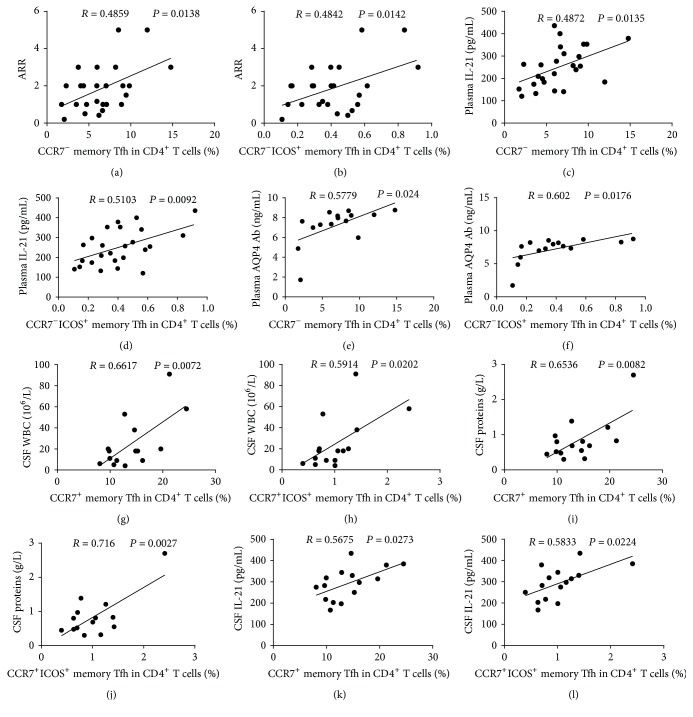
Correlation analysis of different subsets of circulating memory Tfh cells with the values of clinical measures in NMO/NMOSD patients. ((a)-(b)) The percentages of CCR7^−^ and CCR7^−^ICOS^+^ memory Tfh cells were positively associated with the ARR in NMO/NMOSD patients. ((c)-(d)) The percentages of CCR7^−^ and CCR7^−^ICOS^+^ memory Tfh cells were positively associated with the levels of plasma IL-21. ((e)-(f)) The percentages of CCR7^−^ and CCR7^−^ICOS^+^ memory Tfh cells were positively associated with the levels of plasma AQP4 Ab in AQP4 Ab-seropositive patients. ((g)-(h)) The percentages of CCR7^+^ and CCR7^+^ICOS^+^ memory Tfh cells were positively correlated with CSF WBC counts. ((i)-(j)) The percentages of CCR7^+^ and CCR7^+^ICOS^+^ memory Tfh cells were positively correlated with the levels of CSF proteins. ((k)-(l)) The percentages of CCR7^+^ and CCR7^+^ICOS^+^ memory Tfh cells were positively correlated with the levels of CSF IL-21.

In order to understand the potential role of circulating memory Tfh cells, we analyzed the relationships between different subsets of memory Tfh cells and the values of clinical measures tested in the NMO/NMOSD patients. We found that the percentages of CCR7^−^ and CCR7^−^ICOS^+^ memory Tfh cells were positively correlated with the annual relapse rate (ARR; Figures 6(a) and 6(b)) and with the levels of plasma IL-21 in the NMO/NMOSD patients (Figures 6(c) and 6(d)). Among AQP4 Ab-seropositive patients, the percentages of CCR7^−^ and CCR7^−^ICOS^+^ memory Tfh cells were positively correlated with the levels of plasma AQP4 Ab (Figures 6(e) and 6(f)). In addition, the percentages of CCR7^+^ and CCR7^+^ICOS^+^ memory Tfh cells were positively correlated with CSF WBC counts, CSF protein levels, and CSF IL-21 levels in the NMO/NMOSD patients (Figures 6(g)–6(l)). However, there were no correlations among the values of other measures tested.

### 3.6. Treatment with Corticosteroids Reduced the Numbers of Different Subsets of Circulating Memory Tfh Cells and the Levels of Plasma IL-21 in NMO/NMOSD Patients

**Figure 7:**
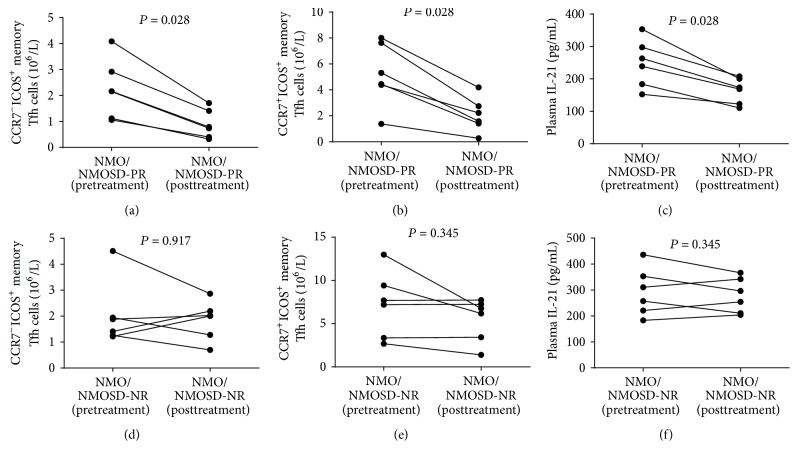
Changes in the numbers of different subsets of circulating memory Tfh cells and plasma IL-21 levels in NMO/NMOSD patients. Among 12 patients with posttreatment follow-up, six patients achieved partial remission (PR) and six patients showed nonremission (NR). The numbers of different subsets of circulating memory Tfh cells and the levels of plasma IL-21 were compared in patients with PR before and after treatment or patients with NR before and after treatment. ((a)-(b)) The numbers of CCR7^−^ICOS^+^, CCR7^+^ICOS^+^ memory Tfh cells in CD4^+^ T cells in patients with PR before and after treatment. (c) The levels of plasma IL-21 in patients with PR before and after treatment. ((d)-(e)) The numbers of CCR7^−^ICOS^+^, CCR7^+^ICOS^+^ memory Tfh cells among CD4^+^ T cells in patients with NR before and after treatment. (f) The levels of plasma IL-21 in patients with NR before and after treatment.

Among 12 patients with posttreatment follow-up, six patients achieved partial remission (PR) and six patients showed nonremission (NR). To further elucidate the role of corticosteroids in memory Tfh cells and the levels of plasma IL-21, we analyzed the numbers of different subsets of circulating memory Tfh cells and the levels of plasma IL-21 before and after treatment in these 12 patients. We found that the numbers of CCR7^−^ICOS^+^ and CCR7^+^ICOS^+^ memory Tfh cells as well as the levels of plasma IL-21 in patients who achieved PR were significantly lower than those before treatment (*P* = 0.028 for all; Figures 7(a)–7(c)). However, no evident change was found in these parameters among patients who showed NR (Figures 7(d)–7(f)).

## 4. Discussion

Tfh cells are crucial for humoral immunity especially antibody production [23]. Tfh cells yield a population of cells that exit the GC and lymphoid tissues and then return to the circulation as a population of quiescent memory-type CD4^+^CXCR5^+^ T cells [18]. A recent study has shown that the percentage of circulating CD4^+^CXCR5^+^PD-1^+^ T cells is higher not only in NMOSD patients than in HCs, but also in patients with relapsing NMOSD than in NMOSD patients in remission [24]. After treatment with methylprednisolone, the CD4^+^CXCR5^+^PD-1^+^ T cell population decreases [24]. These data suggest that Tfh cells may be related to the pathogenesis of NMOSD. However, our study showed that the percentages and numbers of circulating memory Tfh cells and ICOS^+^ memory Tfh cells were higher in NMO/NMOSD patients than in HCs. The difference may be because the sample populations that we studied were both small and heterogeneous. Further studies are needed to clarify the accurate role of PD-1^+^ and ICOS^+^ memory Tfh cells. Circulating memory Tfh cells can provide support for B cell antibody production [25] and be used as a biomarker to monitor the Tfh program in human autoimmune diseases [26]. ICOS is one of the most important surface molecules expressed on Tfh cells, and ICOS supports the differentiation and maintenance of Tfh cell [27]. The absence of ICOS signaling prevents B cell differentiation into memory B cells and GC formation [28]. Hence, ICOS^+^ memory Tfh cells may be vital memory Tfh cells, associated with the relapse of NMO/NMOSD.

Memory T cells consist of two subsets, CCR7^−^ memory (effector memory) T cells and CCR7^+^ memory (central memory) T cells. Upon secondary antigenic stimulation, CCR7^−^ memory T cells can induce immediate protection in peripheral tissues, and CCR7^+^ memory T cells can migrate to secondary lymphoid organs where they have an effector function to antigens [1]. In the current study, the percentages and numbers of CCR7^−^, CCR7^−^ICOS^+^ memory Tfh cells were significantly higher in NMO/NMOSD patients than in HCs. The percentages of CCR7^−^ and CCR7^−^ICOS^+^ memory Tfh cells were positively correlated with the ARR. In addition, among AQP4 Ab-seropositive patients, the percentages of CCR7^−^ and CCR7^−^ICOS^+^ memory Tfh cells were positively correlated with the levels of plasma AQP4 Ab. A recent study found that a high percentage of circulating CCR7^−^PD-1^+^ Tfh cells exists in systemic lupus erythematosus (SLE) patients, which represented active Tfh differentiation in secondary lymphoid tissues and was related to clinical features of autoimmune disease, suggesting that this subset may participate in the pathogenic antibody response in autoimmune diseases [29]. Accordingly, we speculate that CCR7^−^ and CCR7^−^ICOS^+^ memory Tfh cells may participate in the production of plasma AQP4 Ab in peripheral tissues and be related to disease relapse. Moreover, we also found that the percentages and numbers of CCR7^+^ and CCR7^+^ICOS^+^ memory Tfh cells were significantly higher in NMO/NMOSD patients in our study. The percentages of CCR7^+^ and CCR7^+^ICOS^+^ memory Tfh cells were positively correlated with CSF WBC counts and CSF protein levels. It has been shown that circulating CCR7^+^ memory Tfh cells migrate to B cell follicles where they provided help to B cells, representing a distinct memory cell subset specialized in supporting the antibody-mediated immune response [30]. CCR7 is a chemokine receptor required for the migration of T cells and dendritic cells [31]. CCR7 is a potent chemokine signal for controlling CNS entry and migration of lymphocytes in both healthy and diseased states [32–34]. CSF WBC cells, CSF protein levels, and CSF IgG levels in NMO/NMOSD patients were greater than those in NND patients, which reflected CNS inflammation, immune reactions, and blood-brain barrier disruption in NMO/NMOSD patients [35, 36]. Hence, we speculate that CCR7^+^ and CCR7^+^ICOS^+^ memory Tfh cells may circulate into the CNS and contribute to the immune response there. Therefore, CCR7^−^ and CCR7^−^ICOS^+^ memory Tfh cells may participate in AQP4 Ab production in the periphery and be related to the relapse of NMO/NMOSD, whereas CCR7^+^ memory Tfh cells may contribute to immune responses in the CNS of NMO/NMOSD patients.

IL-21, which is the most important cytokine of Tfh cells, plays a critical role in the survival of Tfh cells and the survival, proliferation, and differentiation of GC B cells [37]. Our results showed that the plasma levels of IL-21 were higher in NMO/NMOSD patients, in line with the results of a previous report [24]. Also, the levels of CSF IL-21 were significantly higher in NMO/NMOSD patients than in NND patients. Consistent with this, another study showed that the concentration of CSF IL-21 was elevated and might be positively correlated with humoral activity in NMO [38]. In addition, we found that the percentages of CCR7^−^ and CCR7^−^ICOS^+^ memory Tfh cells were positively correlated with the levels of plasma IL-21. We also found that the percentages of CCR7^+^ and CCR7^+^ICOS^+^ memory Tfh cells were positively correlated with the levels of CSF IL-21 in NMO/NMOSD patients. Hence, IL-21 may participate in the development and relapse of NMO/NMOSD.

Corticosteroids are beneficial to NMO/NMOSD patients with PR based on improvement of the EDSS score. The effect of intravenous methylprednisolone on memory Tfh cells and plasma IL-21 is another interesting finding in our study. The numbers of CCR7^−^ICOS^+^ and CCR7^+^ICOS^+^ memory Tfh cells and the levels of plasma IL-21 significantly decreased in NMO/NMOSD patients with PR, but not in those with NR, suggesting that intravenous methylprednisolone therapy has a suppressive effect on memory Tfh cells. A recent study showed that corticosteroids promote Tfh cell apoptosis by regulating IL-21 and IL-6 levels in SLE patients [14]. These data further indicate that memory Tfh cells may take part in the pathogenic course and relapse of NMO/NMOSD. However, the precise molecular mechanisms by which corticosteroids regulate Tfh cells in NMO/NMOSD require further research.

Surprisingly, we found no differences in the percentages and numbers of different subsets of memory Tfh cells between seropositive and seronegative patients. Other antibodies (such as myelin oligodendrocyte glycoprotein antibodies [8] and AQP1 Ab [39]) exist in seronegative patients. We speculate that memory Tfh cells may participate in the production of other antibodies in AQP4 Ab-seronegative patients. Moreover, among AQP4 Ab-seropositive patients, CCR7^−^ and CCR7^−^ICOS^+^ memory Tfh cells were positively correlated with the levels of plasma AQP4 Ab, whereas CCR7^+^ and CCR7^+^ICOS^+^ memory Tfh cells were not positively correlated with the levels of CSF AQP4 Ab. It is commonly believed that AQP4 Ab is more abundant in the peripheral blood of NMO patients than in their CSF [40]. To date, the origin of CSF AQP4 Ab has not been clarified. More work is necessary to explore the relationship between CCR7^+^ memory Tfh cells and CSF AQP4 Ab and to determine the precise origin of CSF AQP4 Ab.

In summary, our findings provide clear clinical evidence of the relevance of different subsets of circulating memory Tfh cells in relapse and treatment response to corticosteroids among NMO/NMOSD patients. We speculate that circulating memory Tfh cells may participate in the pathogenic course and relapse of NMO/NMOSD. Furthermore, CCR7^−^ and CCR7^−^ICOS^+^ memory Tfh cells may serve as new biomarkers for evaluating disease relapse and may participate in the autoantibody production in the periphery. CCR7^+^ and CCR7^+^ICOS^+^ memory Tfh cells play an instrumental role in the autoimmune inflammation lesions in the CNS. Therefore, memory Tfh cells may provide a new insight into the pathogenesis of NMO/NMOSD as well as a new therapeutic target. There are limitations in this study, such as the small sample size and the lack of long-term follow-up and functional studies of different subsets of memory Tfh cells. Nevertheless, further investigation of the precise role played by memory Tfh cells in NMO/NMOSD pathogenesis and development is warranted.

## Figures and Tables

**Table 1:** The demographic and clinical features of NMO/NMOSD patients and HCs.

	NMO/NMOSD patients	HCs
*n*	25	17
Age (years)	51 (14–63)	47 (18–60)
Female/male	22/3	15/2
ARR	2 (0.2–5)	
Duration of disease (years)	1 (1–20)	
Number of attacks	2 (1–9)	
EDSS scores	3.5 (1–8.5)	
WBC count (10^9^/L)	6.46 (3.83–8.5)	6.4 (3.72–8.91)
Lymphocyte count (10^9^/L)	1.6 (1.14–2.91)	1.72 (1.27–2.29)
AQP4 Ab-seropositive/AQP4 Ab-seronegative	15/10	

Data shown are medians and ranges, except as specified. AQP4 Ab: aquaporin 4 antibody; ARR: annual relapse rate; EDSS: Expanded Disability Status Scale; WBC: white blood cells. Normal values: WBC count, 3.5–9.5 × 10^9^/L; lymphocyte count, 1.1–3.2 × 10^9^/L.

**Table 2:** The demographic and clinical features of NMO/NMOSD and NND patients who received a lumbar puncture.

	NMO/NMOSD patients	NND patients
*n*	15	8
Age (years)	53 (25–63)	39 (25–58)
Female/male	14/1	7/1
Diagnosis	NMO/NMOSD	6-Migraine 2-Idiopathic epilepsy
CSF WBC count (10^6^/L)	18 (4–91)^*∗*^	3 (0–8)
CSF protein (g/L)	0.69 (0.3–2.7)^*∗*^	0.28 (0.18–0.39)
CSF IgG (mg/L)	82.5 (25.3–181)^*∗*^	23 (12–31)

Data shown are medians and ranges, except as specified. CSF: cerebrospinal fluid; NND: noninflammatory neurological disease; WBC: white blood cell. Normal values: CSF WBC count, 0–8 × 10^6^/L; CSF protein concentration, 0.15–0.45 g/L; CSF IgG concentration, 0–34 mg/L. ^*∗*^ *P* < 0.05 versus data for HCs.

**Table 3:** The demographic and clinical features of 12 NMO/NMOSD patients after treatment.

	NMO/NMOSD patients with PR	NMO/NMOSD patients with NR
*n*	6	6
Age (years)	47.5 (25–56)	56 (53–63)
Female/male	5/1	6/0
CSF WBC count (10^6^/L)	19 (5–91)	28 (4–58)
CSF protein (g/L)	0.82 (0.45–1.21)	0.745 (0.52–2.7)
CSF IgG (mg/L)	96 (25.3–176)	88.45 (31.9–181)

Data shown are medians and ranges, except as specified. CR: complete remission; CSF: cerebrospinal fluid; PR: partial remission; WBC: white blood cell. Normal values: CSF WBC count, 0–8 × 10^6^/L; CSF protein concentration, 0.15–0.45 g/L; CSF IgG concentration, 0–34 mg/L.
